# Spontaneous Massive Retinal Pigment Epithelium Tear: A Case Report of a Dramatic Complication of Age-Related Macular Degeneration

**DOI:** 10.7759/cureus.52980

**Published:** 2024-01-26

**Authors:** Renato Correia Barbosa, Carla Teixeira

**Affiliations:** 1 Ophthalmology, Hospital Pedro Hispano, Unidade Local de Saúde de Matosinhos (ULSM), Matosinhos, PRT

**Keywords:** anti-vegf treatment, retinal hemorrhage, age-related macular degeneration (amd), retinal pigment epithelium tear, retinal pigment epithelium (rpe)

## Abstract

Retinal pigment epithelium (RPE) tears occur when the RPE acutely breaks and retracts, leaving the underlying Bruch’s membrane and choroid exposed. They usually happen in areas of previous pigment epithelial detachments and are generally associated with age-related macular degeneration (AMD). The purpose of this report is to describe a case of a spontaneous massive central RPE tear in a patient with untreated AMD.

A 67-year-old female patient presented with complaints of sudden decreased vision in her right eye. Her best-corrected visual acuity was 2/20, and fundoscopy revealed a massive central retinal hemorrhage with intraretinal, subretinal, and sub-RPE blood. The patient started anti-vascular endothelial growth factor (VEGF) treatment, and after the blood was reabsorbed, a very large central tear of the RPE involving the central macula was evident, with a layer of detached retina folded on itself. She received continuous anti-VEGF therapy, and the final measurement of her visual acuity was 2/200, despite the complete reabsorption of the hemorrhage.

RPE tears may occur spontaneously as part of the natural history of AMD or be triggered by the initiation of anti-VEGF treatment in the presence of large pigment epithelium detachments. There are currently no strategies to prevent their spontaneous development, and they constitute a dramatic complication of AMD. The prognosis is dependent on the size and location of the lesion, and the visual loss is irreversible.

## Introduction

Retinal pigment epithelium (RPE) tears were first described by Hoskin et al. in 1981 [1] and occur when the RPE acutely breaks and retracts in an area of the retina, leaving the underlying Bruch’s membrane and choroid exposed. They usually happen in areas overlying a pigment epithelial detachment (PED), between the region of detached and flat RPE. They consist of serious events that are associated with severe vision loss, especially if the central macula is affected.

RPE tears are typically seen in patients with neovascular age-related macular degeneration (AMD), as a natural history of the disease, or as a response to laser or anti-vascular endothelial growth factor (VEGF) treatment for choroidal neovascularization. They have been reported in various chorioretinal diseases such as central serous chorioretinopathy, polypoidal choroidal vasculopathy, trauma, pathologic myopia, and proliferative vitreoretinopathy, among others [2-3], but their incidence is relatively rare, with most studies focusing on AMD patients.

While they are frequently linked to anti-VEGF intravitreal treatments, they are part of the natural history of AMD, and the most important risk factor for their development is the presence of PEDs, which consist of the separation of the RPE from the underlying Bruch’s membrane with serous fluid, blood, drusenoid material, or combinations of those. Bird hypothesized that the mechanism for the development of tearing seems to be related to the hydrostatic pressure from the fluid accumulated inside the PED, which eventually causes the rupture of the RPE [4]. More recent theories propose that RPE tears form due to the contraction of type 1 neovascular membranes under the RPE.

The purpose of this report is to describe the case of a female patient with previously known intermediate AMD who developed a spontaneous massive central RPE tear with an irreversible and profound impact on visual acuity.

## Case presentation

**Figure 1:**
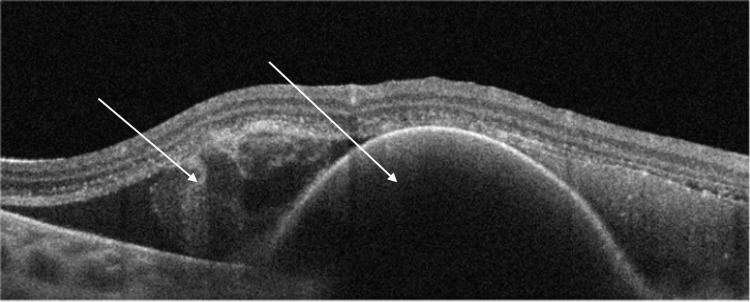
Spectral-domain optical coherence tomography (SD-OCT) upon presentation, showing a large collection of subretinal and subretinal pigment epithelium (RPE) blood

A 67-year-old female patient presented in the emergency department with complaints of vision in the right eye (OD) for three days. She had a history of bilateral cataract surgery three years prior and OD pterygium surgery four years before. Previous visits revealed the presence of mild bilateral macular pigmentary changes and macular drusen, compatible with intermediate AMD. During the examination, the patient had a best corrected visual acuity (BCVA) of 2/20 in her OD with eccentric fixation. The anterior segment slit-lamp exam was unremarkable, and fundoscopy under pharmacological mydriasis showed a severe macular hemorrhage on OD. Spectral-domain optical coherence tomography (SD-OCT) showed intraretinal, subretinal, and sub-RPE blood (Figure 1).

The left eye (OS) showed pigmentary changes and macular drusens, compatible with intermediate AMD. A pars plana vitrectomy with subretinal injection of alteplase was immediately considered, but this hypothesis was ruled out since most of the blood was in the sub-RPE position. The patient was proposed to receive intravitreal injections (IVI) of eflibercept (Eylea).

**Figure 2:**
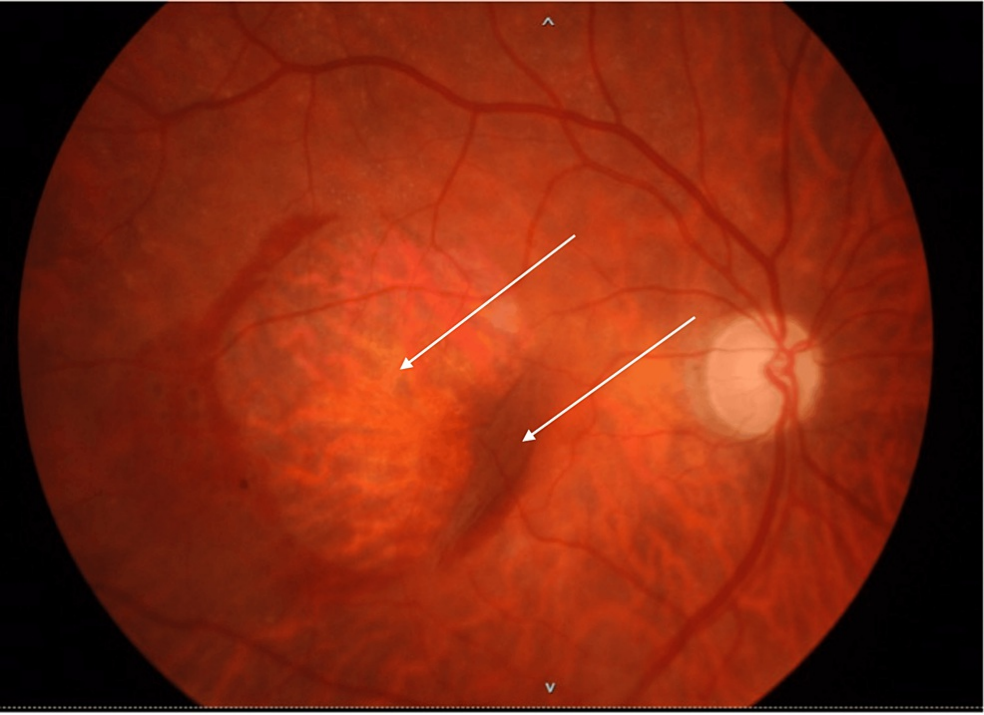
Color fundus photograph after six months, showing exposed Bruch’s membrane and choroid, and a large retinal and retinal pigment epithelium (RPE) fold

**Figure 3:**
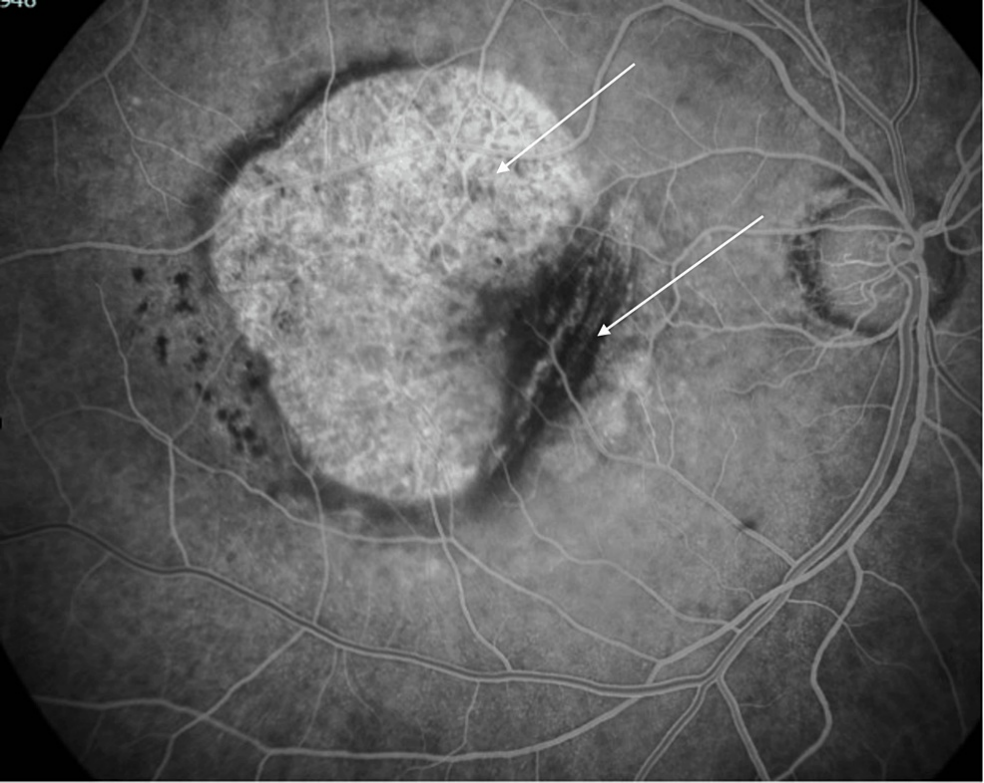
Red-free fundus photograph after six months, showing exposed Bruch's membrane and choroid, and a large retinal and retinal pigment epithelium (RPE) fold

**Figure 4:**
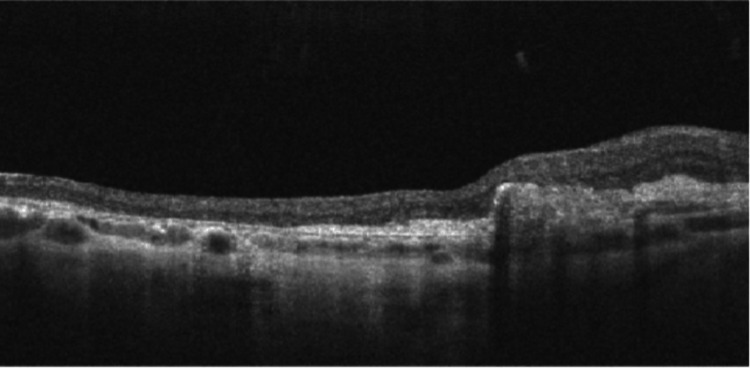
Spectral-domain optical coherence tomography (SD-OCT) of the macula, one year after the initial presentation

Three months later, after three doses of aflibercept IVI, the patient still had a significant macular hemorrhage. Additionally, a very large central tear of the RPE was now evident, occupying practically the entire region of the central macula, as well as a layer of detached retina folded under itself in the nasal macula. SD-OCT was repeated, and a fluorescein angiography was performed, which corroborated the findings. The patient maintained IVI treatment with aflibercept monthly for the first six months and then every two months. She was then followed up every three months until the macular hemorrhage resolved completely. The final appearance of the central macula showed exposed Bruch's membrane and choroidal tissue, with the retina + RPE complex torn into itself in the nasal macula (Figures 2-4). The final OD BCVA was 2/200.

## Discussion

RPE tears are one of the most dramatic complications of the natural history of AMD, happening as part of the natural history of PEDs or as a complication of the treatment with anti-VEGF therapy. Given the exponential increase of IVIs for the treatment of AMD during the last decades, there has been a marked increase in the incidence of RPE tears. Vazquez et al. showed that this complication may occur in 10-12.5% of patients as part of the natural history of AMD, as opposed to 14-19.7% after anti-VEGF injections [5], which translates to a significant difference given the very high number of patients under anti-VEGF treatment.

Knowing that a significant proportion of patients, either spontaneously or as a complication of the treatment, will develop this complication, and given its profound irreversible effect on the patient’s visual function, studies in recent years have focused on the factors that may predict a higher risk for its development. The most important risk factor seems to be the size of the pre-existing PEDs. Sarraf et al. showed that PEDs with a vertical height of more than 550 µm pose a higher risk of rupture [6], while Chan et al. considered this cut-off to be at 400 µm [7]. In addition to height, the surface area and the internal diameter of the base of the PED are also related to a higher risk of complications [8]. Additionally, the morphological characteristics of the PEDs that could predict the risk of degeneration were also investigated. Sarraf et al. stated that the existence of vascularized PEDs with the angiographic “ring sign," which shows hyperfluorescence at the edges of the lesions, could indicate micro-ruptures, which may translate into a higher risk of RPE macrorupture at the time anti-VEGF treatment is started [9]. On the other hand, Doguizi and Ozdek demonstrated an inverse relationship between the duration of PEDs and the occurrence of RPE tears, characterizing several cases in which the recent appearance of PEDs was associated with a higher incidence of tears in patients who started anti-VEGF therapy. The authors justified this relationship by the fact that a recent PED represents a recent neovascular process, with immature vessels that are more likely to contract abruptly upon the action of an anti-VEGF drug [10].

Since RPE tears are part of the natural history of AMD, there are no strategies to prevent their spontaneous development in most cases. Given their relationship with large PEDs, patients should be counseled about the possibility of a sudden vision loss event, especially if this occurs following an IVI, despite there not really being any treatment that can be made after their onset. Some authors suggest discontinuing intravitreal treatment when high-risk factors are identified, but there is little consensus on this approach since the risk of complications is part of the natural history of the disease, regardless of whether the treatment is carried out [10-11]. There are currently no conclusions regarding the relative risk between the different anti-VEGF drugs.

The main reason for the severity of this disease is the fact that after its occurrence, there are no alternatives for visual rehabilitation since the entire tissue of the neurosensory retina and the RPE itself lose their viability and are often even folded on themselves, as in our clinical case. The size and centrality of the tear worsen the prognosis, as a larger and more central area of affected tissue translates into a bigger amputation of the central visual field and a consequently bigger central scotoma, which provides a major handicap for daily activities. The therapeutic approach is to monitor the patient over the long term. Maintenance treatment with anti-VEGFs may be beneficial, given the fact that, in most cases, they constitute the treatment of the underlying disease responsible for this complication. No long-term benefit has been proven for discontinuing anti-VEGF treatment after the RPE tear occurred, so maintenance therapy with IVI is the generally employed strategy [11]. As mentioned, the long-term visual prognosis is entirely dependent on the location and size of the tear. Small paracentral lesions may have a minimal impact on the patient’s visual function, but large foveal lesions are generally associated with irreversible central visual loss. In the presented case, a very large and central tear makes any future treatment unfeasible, with severe irreversible vision loss.

## Conclusions

This clinical case shows that even in the absence of previous anti-VEGF treatment, the natural history of AMD may lead to the formation of large central RPE tears, which translates into a very poor visual prognosis in the long term. At present, there are no valid strategies for the prevention or targeted treatment of this devastating complication. Therefore, ongoing research is necessary to improve treatment options and improve patients' quality of life.
